# Correction: Cognitive effort investment: Does disposition become action?

**DOI:** 10.1371/journal.pone.0326508

**Published:** 2025-06-13

**Authors:** Corinna Kührt, Sven-Thomas Graupner, Philipp C. Paulus, Alexander Strobel

There are errors in the Funding statement. The correct Funding statement is as follows: This research and the Article Processing Charge (APC) were funded by the German Research Foundation (Deutsche Forschungsgemeinschaft, DFG; SFB 940/2 project B6). The funders had no role in study design, data collection and analysis, decision to publish, or preparation of the manuscript.

## References

[1] Kührt C, Graupner S-T, Paulus PC, Strobel A. Cognitive effort investment: Does disposition become action?. PLoS One. 2023;18(8):e0289428. doi: 10.1371/journal.pone.0289428 37607171 PMC10443884

