# Supplementary material for: Cognitive effort investment: Does disposition become action?
Source: PLoS One. 2023 Aug 22;18(8):e0289428. doi: 10.1371/journal.pone.0289428 (PMC10443884; doi:10.1371/journal.pone.0289428)
Supplement: S1 Table — Note. N ≥ 144. ***p < .001, **p < .01, *p < .05. COM = cognitive motivation, ESC = effortful self-control. (DOCX) [file pone.0289428.s001.docx]

**S1 Table.** **Results for LMM and GLMM analyses on first-order factors for *n*-back and flanker task.**

|  |  | ***N*-back** | | | | |  | **Flanker** | | | | |
| --- | --- | --- | --- | --- | --- | --- | --- | --- | --- | --- | --- | --- |
|  | Parameter | Beta | SE | *p*-value | | Random Effects (SD) |  | Beta | SE | *p*-value | | Random Effects (SD) |
| *Cognitive Motivation* | |  |  |  |  |  |  |  |  |  |  |  |
| **Perceived Task Load** | Intercept | -0.42 | 0.06 | <.001 | *** | 0.73 |  |  |  |  |  |  |
|  | Demand | 0.45 | 0.03 | <.001 | *** | 0.30 |  |  |  |  |  |  |
|  | COM | -0.07 | 0.08 | .418 |  |  |  |  |  |  |  |  |
|  | COM × Demand | -0.02 | 0.04 | .667 |  |  |  |  |  |  |  |  |
| **Reaction Time** | Intercept | 547.66 | 6.29 | <.001 | *** | 76.34 |  | 348.76 | 4.12 | <.001 | *** | 49.93 |
|  | Demand | 83.40 | 4.94 | <.001 | *** | 59.6 |  | 13.43 | 0.58 | <.001 | *** | 6.54 |
|  | Payoff | 0.37 | 0.77 | .636 |  | 8.08 |  | -0.78 | 0.37 | .037 | * | 4.11 |
|  | COM | 2.38 | 8.39 | .777 |  |  |  | 6.36 | 5.52 | .251 |  |  |
|  | Demand × Payoff | 1.05 | 1.17 | .372 |  | 12.94 |  | -0.15 | 0.16 | .342 |  | 0.79 |
|  | COM × Demand | 3.09 | 6.56 | .638 |  |  |  | 0.87 | 0.76 | .255 |  |  |
|  | COM × Payoff | 3.12 | 1.03 | .003 | ** |  |  | 1.25 | 0.50 | .013 | * |  |
|  | COM × Demand × Payoff | 3.36 | 1.55 | .032 | * |  |  | -0.13 | 0.22 | .538 |  |  |
| **Accuracy** | Intercept | 3.63 | 0.06 | <.001 | *** | 0.68 |  | 3.55 | 0.09 | <.001 | *** | 0.98 |
|  | Demand | -0.82 | 0.05 | <.001 | *** | 0.42 |  | -1.32 | 0.04 | <.001 | *** | 0.43 |
|  | Payoff | -0.01 | 0.02 | .773 |  | 0.01 |  | 0.03 | 0.03 | .192 |  | 0.14 |
|  | COM | 0.14 | 0.08 | .094 |  |  |  | 0.07 | 0.11 | .518 |  |  |
|  | Demand × Payoff | 0.02 | 0.02 | .500 |  | 0.13 |  | 0.00 | 0.01 | .931 |  | 0.08 |
|  | COM × Demand | 0.05 | 0.06 | .383 |  |  |  | 0.01 | 0.05 | .797 |  |  |
|  | COM × Payoff | 0.00 | 0.02 | .838 |  |  |  | -0.01 | 0.03 | .673 |  |  |
|  | COM × Demand × Payoff | -0.03 | 0.03 | .361 |  |  |  | 0.00 | 0.02 | .833 |  |  |
| **Early Frontal Midline Theta Power late Frontal Midline Theta Power** | Intercept | 2.27 | 0.13 | <.001 | *** | 1.58 |  | 2.41 | 0.14 | <.001 | *** | 1.68 |
|  | Demand | -0.16 | 0.05 | .001 | ** | 0.50 |  | 0.47 | 0.06 | <.001 | *** | 0.73 |
|  | Payoff | 0.03 | 0.02 | .091 |  | 0.09 |  | -0.01 | 0.02 | .659 |  | 0.13 |
|  | COM | 0.16 | 0.18 | .363 |  |  |  | 0.21 | 0.19 | .273 |  |  |
|  | Demand × Payoff | 0.00 | 0.02 | .842 |  | 0.14 |  | 0.00 | 0.02 | .947 |  | 0.14 |
|  | COM × Demand | 0.06 | 0.06 | .323 |  |  |  | 0.15 | 0.08 | .077 |  |  |
|  | COM × Payoff | -0.03 | 0.02 | .260 |  |  |  | -0.01 | 0.03 | .758 |  |  |
|  | COM × Demand × Payoff | -0.02 | 0.03 | .495 |  |  |  | -0.04 | 0.03 | .128 |  |  |
| **Late Frontal Midline Theta Power** | Intercept | 0.54 | 0.06 | <.001 | *** | 0.66 |  | 0.55 | 0.08 | <.001 | *** | 0.94 |
|  | Demand | 0.02 | 0.05 | .692 |  | 0.56 |  | -0.07 | 0.03 | .019 | * | 0.24 |
|  | Payoff | 0.01 | 0.15 | .936 |  | 1.76 |  | -0.02 | 0.58 | .970 |  | 7.04 |
|  | COM | -0.10 | 0.08 | .186 |  |  |  | -0.20 | 0.11 | .061 |  |  |
|  | Demand × Payoff | 0.00 | 0.11 | .980 |  | 1.35 |  | -0.02 | 0.24 | .930 |  | 2.87 |
|  | COM × Demand | 0.10 | 0.07 | .126 |  |  |  | 0.03 | 0.04 | .440 |  |  |
|  | COM × Payoff | 0.02 | 0.19 | .906 |  |  |  | 0.01 | 0.77 | .991 |  |  |
|  | COM × Demand × Payoff | -0.02 | 0.15 | .905 |  |  |  | -0.01 | 0.32 | .966 |  |  |
| **N2 Amplitude** | Intercept | -0.67 | 0.17 | <.001 | *** | 2.05 |  | 0.48 | 0.19 | .011 | * | 2.26 |
|  | Demand | 0.61 | 0.04 | <.001 | *** | 0.45 |  | -0.26 | 0.04 | <.001 | *** | 0.45 |
|  | Payoff | 0.01 | 0.02 | .499 |  | 0.10 |  | 0.03 | 0.03 | .283 |  | 0.26 |
|  | COM | 0.13 | 0.23 | .578 |  |  |  | 0.10 | 0.25 | .704 |  |  |
|  | Demand × Payoff | 0.00 | 0.02 | .897 |  | 0.15 |  | 0.01 | 0.02 | .552 |  | 0.10 |
|  | COM × Demand | -0.08 | 0.06 | .134 |  |  |  | -0.08 | 0.05 | .126 |  |  |
|  | COM × Payoff | 0.01 | 0.02 | .749 |  |  |  | 0.05 | 0.03 | .156 |  |  |
|  | COM × Demand × Payoff | -0.01 | 0.03 | .821 |  |  |  | 0.01 | 0.02 | .508 |  |  |
| **P3 Amplitude** | Intercept | 4.58 | 0.16 | <.001 | *** | 1.97 |  | 5.10 | 0.18 | <.001 | *** | 2.13 |
|  | Demand | -0.60 | 0.07 | <.001 | *** | 0.77 |  | 0.15 | 0.06 | .011 | * | 0.64 |
|  | Payoff | 0.03 | 0.02 | .122 |  | 0.16 |  | 0.08 | 0.02 | <.001 | *** | 0.19 |
|  | COM | -0.07 | 0.22 | .760 |  |  |  | 0.06 | 0.24 | .811 |  |  |
|  | Demand × Payoff | -0.02 | 0.02 | .405 |  | 0.14 |  | -0.01 | 0.01 | .643 |  | 0.05 |
|  | COM × Demand | -0.10 | 0.09 | .254 |  |  |  | -0.06 | 0.07 | .404 |  |  |
|  | COM × Payoff | -0.05 | 0.03 | .051 |  |  |  | 0.02 | 0.03 | .560 |  |  |
|  | COM × Demand × Payoff | -0.02 | 0.03 | .393 |  |  |  | 0.00 | 0.02 | .879 |  |  |
| **Pupil Dilation** | Intercept | 52.18 | 2.59 | <.001 | *** | 31.03 |  | 60.9 | 3.01 | <.001 | *** | 35.86 |
|  | Demand | 0.17 | 1.29 | .894 |  | 14.70 |  | 8.57 | 0.75 | <.001 | *** | 7.52 |
|  | Payoff | 0.15 | 0.35 | .671 |  | 2.40 |  | 1.80 | 0.51 | .001 | ** | 4.87 |
|  | COM | 2.70 | 3.46 | .436 |  |  |  | 0.74 | 4.02 | .854 |  |  |
|  | Demand × Payoff | -0.31 | 0.44 | .490 |  | 3.36 |  | 0.34 | 0.34 | .325 |  | 2.15 |
|  | COM × Demand | -1.13 | 1.71 | .510 |  |  |  | 0.71 | 0.96 | .465 |  |  |
|  | COM × Payoff | -0.16 | 0.46 | .721 |  |  |  | 0.11 | 0.67 | .868 |  |  |
|  | COM × Demand × Payoff | -0.60 | 0.59 | .314 |  |  |  | -0.10 | 0.46 | .832 |  |  |
| *Effortful Self-Control* | |  |  |  |  |  |  |  |  |  |  |  |
| **Perceived Task Load** | Intercept | -0.42 | 0.06 | <.001 | *** | 0.72 |  |  |  |  |  |  |
|  | Demand | 0.45 | 0.03 | <.001 | *** | 0.30 |  |  |  |  |  |  |
|  | ESC | -0.21 | 0.09 | .014 | * |  |  |  |  |  |  |  |
|  | ESC × Demand | -0.02 | 0.04 | .564 |  |  |  |  |  |  |  |  |
| **Reaction Time** | Intercept | 547.66 | 6.29 | <.001 | *** | 76.32 |  | 348.76 | 4.12 | <.001 | *** | 49.93 |
|  | Demand | 83.40 | 4.94 | <.001 | *** | 59.63 |  | 13.43 | 0.58 | <.001 | *** | 6.57 |
|  | Payoff | 0.37 | 0.79 | .641 |  | 8.27 |  | -0.78 | 0.37 | .037 | * | 4.13 |
|  | ESC | -2.68 | 8.78 | .760 |  |  |  | -4.86 | 5.78 | .402 |  |  |
|  | Demand × Payoff | 1.05 | 1.19 | .377 |  | 13.16 |  | -0.15 | 0.16 | .337 |  | 0.72 |
|  | ESC × Demand | -1.46 | 6.83 | .832 |  |  |  | 0.49 | 0.79 | .536 |  |  |
|  | ESC × Payoff | 2.20 | 1.09 | .045 | * |  |  | 1.20 | 0.52 | .022 | * |  |
|  | ESC × Demand × Payoff | 1.22 | 1.64 | .457 |  |  |  | -0.47 | 0.22 | .037 | * |  |
| **Accuracy** | Intercept | 3.63 | 0.06 | <.001 | *** | 0.67 |  | 3.55 | 0.09 | <.001 | *** | 0.98 |
|  | Demand | -0.82 | 0.05 | <.001 | *** | 0.42 |  | -1.32 | 0.04 | <.001 | *** | 0.43 |
|  | Payoff | -0.01 | 0.02 | .769 |  | 0.01 |  | 0.03 | 0.03 | .185 |  | 0.13 |
|  | ESC | 0.20 | 0.08 | .018 | * |  |  | 0.09 | 0.12 | .453 |  |  |
|  | Demand × Payoff | 0.02 | 0.02 | .514 |  | 0.13 |  | 0.00 | 0.01 | .914 |  | 0.08 |
|  | ESC × Demand | -0.03 | 0.06 | .634 |  |  |  | -0.04 | 0.05 | .408 |  |  |
|  | ESC × Payoff | -0.02 | 0.02 | .462 |  |  |  | 0.03 | 0.03 | .257 |  |  |
|  | ESC × Demand × Payoff | -0.02 | 0.03 | .477 |  |  |  | -0.02 | 0.02 | .309 |  |  |
| **Early Frontal Midline Theta Power late Frontal Midline Theta Power** | Intercept | 2.27 | 0.13 | <.001 | *** | 1.56 |  | 2.41 | 0.14 | <.001 | *** | 1.66 |
|  | Demand | -0.16 | 0.05 | .001 | ** | 0.50 |  | 0.47 | 0.06 | <.001 | *** | 0.73 |
|  | Payoff | 0.03 | 0.02 | .092 |  | 0.09 |  | -0.01 | 0.02 | .658 |  | 0.13 |
|  | ESC | 0.36 | 0.18 | .050 |  |  |  | 0.38 | 0.20 | .054 |  |  |
|  | Demand × Payoff | 0.00 | 0.02 | .842 |  | 0.14 |  | 0.00 | 0.02 | .953 |  | 0.13 |
|  | ESC × Demand | -0.06 | 0.07 | .344 |  |  |  | 0.18 | 0.09 | .039 | * |  |
|  | ESC × Payoff | -0.01 | 0.02 | .675 |  |  |  | -0.01 | 0.03 | .655 |  |  |
|  | ESC × Demand × Payoff | -0.02 | 0.03 | .503 |  |  |  | -0.06 | 0.03 | .019 | * |  |
| **Late Frontal Midline Theta Power** | Intercept | 0.54 | 0.05 | <.001 | *** | 0.63 |  | 0.55 | 0.08 | <.001 | *** | 0.91 |
|  | Demand | 0.02 | 0.24 | .928 |  | 2.92 |  | -0.07 | 0.03 | .018 | * | 0.24 |
|  | Payoff | 0.01 | 0.02 | .687 |  | 0.17 |  | -0.02 | 0.54 | .967 |  | 41.92 |
|  | ESC | -0.07 | 0.08 | .348 |  |  |  | -0.15 | 0.11 | .187 |  |  |
|  | Demand × Payoff | 0.00 | 0.38 | .991 |  | 4.64 |  | -0.02 | 0.04 | .751 |  | 0.49 |
|  | ESC × Demand | -0.02 | 0.34 | .945 |  |  |  | -0.03 | 0.04 | .451 |  |  |
|  | ESC × Payoff | -0.01 | 0.03 | .816 |  |  |  | 0.01 | 0.75 | .988 |  |  |
|  | ESC × Demand × Payoff | -0.03 | 0.54 | .949 |  |  |  | -0.02 | 0.06 | .849 |  |  |
| **N2 Amplitude** | Intercept | -0.67 | 0.17 | <.001 | *** | 2.04 |  | 0.49 | 0.19 | .011 | * | 2.26 |
|  | Demand | 0.61 | 0.04 | <.001 | *** | 0.45 |  | -0.26 | 0.04 | <.001 | *** | 0.45 |
|  | Payoff | 0.01 | 0.02 | .498 |  | 0.10 |  | 0.03 | 0.03 | .288 |  | 0.26 |
|  | ESC | 0.18 | 0.24 | .465 |  |  |  | 0.24 | 0.27 | .369 |  |  |
|  | Demand × Payoff | 0.00 | 0.02 | .899 |  | 0.15 |  | 0.01 | 0.02 | .547 |  | 0.10 |
|  | ESC × Demand | 0.00 | 0.06 | .939 |  |  |  | -0.07 | 0.06 | .249 |  |  |
|  | ESC × Payoff | 0.01 | 0.02 | .782 |  |  |  | 0.01 | 0.04 | .810 |  |  |
|  | ESC × Demand × Payoff | -0.04 | 0.03 | .196 |  |  |  | 0.03 | 0.02 | .128 |  |  |
| **P3 Amplitude** | Intercept | 4.58 | 0.16 | <.001 | *** | 1.97 |  | 5.10 | 0.18 | <.001 | *** | 2.13 |
|  | Demand | -0.60 | 0.07 | <.001 | *** | 0.76 |  | 0.15 | 0.06 | .011 | * | 0.64 |
|  | Payoff | 0.03 | 0.02 | .122 |  | 0.16 |  | 0.08 | 0.02 | <.001 | *** | 0.19 |
|  | ESC | -0.04 | 0.23 | .872 |  |  |  | 0.01 | 0.25 | .973 |  |  |
|  | Demand × Payoff | -0.02 | 0.02 | .409 |  | 0.15 |  | -0.01 | 0.01 | .643 |  | 0.05 |
|  | ESC × Demand | -0.11 | 0.09 | .240 |  |  |  | -0.01 | 0.08 | .892 |  |  |
|  | ESC × Payoff | -0.06 | 0.03 | .024 | * |  |  | -0.01 | 0.03 | .666 |  |  |
|  | ESC × Demand × Payoff | 0.00 | 0.03 | .978 |  |  |  | 0.00 | 0.02 | .819 |  |  |
| **Pupil Dilation** | Intercept | 52.16 | 2.59 | <.001 | *** | 31.06 |  | 60.88 | 3.01 | <.001 | *** | 35.79 |
|  | Demand | 0.18 | 1.29 | .889 |  | 14.71 |  | 8.56 | 0.75 | <.001 | *** | 7.53 |
|  | Payoff | 0.15 | 0.34 | .670 |  | 2.39 |  | 1.79 | 0.51 | .001 | ** | 4.86 |
|  | ESC | -2.12 | 3.62 | .559 |  |  |  | -3.27 | 4.18 | .436 |  |  |
|  | Demand × Payoff | -0.31 | 0.45 | .493 |  | 3.40 |  | 0.34 | 0.34 | .322 |  | 2.10 |
|  | ESC × Demand | -0.10 | 1.78 | .955 |  |  |  | -0.19 | 1.00 | .850 |  |  |
|  | ESC × Payoff | 0.20 | 0.47 | .668 |  |  |  | -0.38 | 0.69 | .586 |  |  |
|  | ESC × Demand × Payoff | -0.53 | 0.61 | .386 |  |  |  | -0.71 | 0.47 | .135 |  |  |

*Note*. *N* ≥ 144. ****p* < .001, ***p* < .01, **p* < .05. COM = cognitive motivation, ESC = effortful self-control.
