# Supplementary material for: Cognitive effort investment: Does disposition become action?
Source: PLoS One. 2023 Aug 22;18(8):e0289428. doi: 10.1371/journal.pone.0289428 (PMC10443884; doi:10.1371/journal.pone.0289428)
Supplement: S2 Table — Note. N ≥ 144. ***p < .001, **p < .01, *p < .05. CEI = cognitive effort investment. (DOCX) [file pone.0289428.s002.docx]

**S2 Table.** **Simple slopes analysis results for all effort indices in *n*-back and flanker task.**

|  |  |  | ***N*-back** | | | |  | **Flanker** | | | |
| --- | --- | --- | --- | --- | --- | --- | --- | --- | --- | --- | --- |
|  |  | Value of CEI | Beta (SE) | | 95% CI | Conditional Intercept Beta (SE) |  | Beta (SE) | | 95% CI | Conditional Intercept Beta (SE) |
| **Perceived Task Load** |  | - 1 SD | 0.47 (0.04) | *** | [0.38, 0.55] | -0.31 (0.09) |  |  | |  |  |
|  | Demand | Mean | 0.45 (0.03) | *** | [0.39, 0.51] | -0.42 (0.06) |  |  | |  |  |
|  |  | + 1 SD | 0.43 (0.04) | *** | [0.35, 0.51] | -0.54 (0.09) |  |  | |  |  |
| **Reaction Time** |  | - 1 SD | 82.53 (6.99) | *** | [68.84, 96.23] | 547.60 (8.93) |  | 12.83 (0.81) | *** | [11.23, 14.42] | 347.67 (5.88) |
|  | Demand | Mean | 83.40 (4.94) | *** | [73.72, 93.09] | 547.66 (6.30) |  | 13.43 (0.58) | *** | [12.29, 14.56] | 348.76 (4.14) |
|  |  | + 1 SD | 84.27 (6.99) | *** | [70.58, 97.97] | 547.72 (8.93) |  | 14.03 (0.81) | *** | [12.43, 15.62] | 349.84 (5.88) |
|  |  | - 1 SD | -1.95 (1.09) |  | [-4.10, 0.19] | 547.60 (8.93) |  | -1.84 (0.52) | *** | [-2.86, -0.81] | 347.67 (5.88) |
|  | Payoff | Mean | 0.37 (0.77) |  | [-1.15, 1.89] | 547.66 (6.30) |  | -0.78 (0.37) | ** | [-1.51, -0.06] | 348.76 (4.14) |
|  |  | + 1 SD | 2.69 (1.09) | ** | [0.54, 4.83] | 547.72 (8.93) |  | 0.27 (0.52) |  | [-0.76, 1.30] | 349.84 (5.88) |
| **Accuracy** |  | - 1 SD | -0.84 (0.06) | *** | [-0.96, -0.72] | 3.49 (0.09) |  | -1.31 (0.05) | *** | [-1.41, -1.20] | 3.48 (0.12) |
|  | Demand | Mean | -0.82 (0.05) | *** | [-0.91, -0.74] | 3.63 (0.06) |  | -1.32 (0.04) | *** | [-1.40, -1.24] | 3.55 (0.09) |
|  |  | + 1 SD | -0.81 (0.06) | *** | [-0.93, -0.69] | 3.77 (0.09) |  | -1.33 (0.06) | *** | [-1.44, -1.22] | 3.62 (0.12) |
|  |  | - 1 SD | 0.00 (0.02) |  | [-0.04, 0.05] | 3.49 (0.09) |  | 0.03 (0.03) |  | [-0.04, 0.09] | 3.48 (0.12) |
|  | Payoff | Mean | -0.01 (0.02) |  | [-0.04, 0.03] | 3.63 (0.06) |  | 0.03 (0.03) |  | [-0.02, 0.08] | 3.55 (0.09) |
|  |  | + 1 SD | -0.01 (0.03) |  | [-0.06, 0.04] | 3.77 (0.09) |  | 0.04 (0.03) |  | [-0.03, 0.11] | 3.62 (0.12) |
| **Early Frontal Midline Theta Power** |  | - 1 SD | -0.16 (0.07) | ** | [-0.29, -0.03] | 2.05 (0.19) |  | 0.33 (0.09) | *** | [0.15, 0.50] | 2.16 (0.20) |
|  | Demand | Mean | -0.16 (0.05) | *** | [-0.25, -0.07] | 2.27 (0.13) |  | 0.47 (0.06) | *** | [0.34, 0.59] | 2.41 (0.14) |
|  |  | + 1 SD | -0.15 (0.07) | ** | [-0.29, -0.02] | 2.48 (0.19) |  | 0.61 (0.09) | *** | [0.43, 0.79] | 2.65 (0.20) |
|  |  | - 1 SD | 0.04 (0.02) |  | [0.00, 0.09] | 2.05 (0.19) |  | 0.00 (0.03) |  | [-0.05, 0.05] | 2.16 (0.20) |
|  | Payoff | Mean | 0.03 (0.02) |  | [0.00, 0.06] | 2.27 (0.13) |  | -0.01 (0.02) |  | [-0.05, 0.03] | 2.41 (0.14) |
|  |  | + 1 SD | 0.01 (0.02) |  | [-0.03, 0.06] | 2.48 (0.19) |  | -0.02 (0.03) |  | [-0.07, 0.04] | 2.65 (0.20) |
| **late Frontal Midline Theta Power** |  | - 1 SD | -0.02 (0.07) |  | [-0.15, 0.11] | 0.62 (0.07) |  | -0.07 (0.04) |  | [-0.16, 0.02] | 0.70 (0.12) |
|  | Demand | Mean | 0.02 (0.05) |  | [-0.07, 0.11] | 0.54 (0.05) |  | -0.07 (0.03) | ** | [-0.13, -0.01] | 0.55 (0.08) |
|  |  | + 1 SD | 0.06 (0.07) |  | [-0.07, 0.19] | 0.47 (0.07) |  | -0.07 (0.04) |  | [-0.15, 0.02] | 0.40 (0.12) |
|  |  | - 1 SD | 0.00 (0.02) |  | [-0.04, 0.04] | 0.62 (0.07) |  | -0.03 (0.02) |  | [-0.08, 0.02] | 0.70 (0.12) |
|  | Payoff | Mean | 0.01 (0.02) |  | [-0.02, 0.04] | 0.54 (0.05) |  | -0.02 (0.02) |  | [-0.06, 0.01] | 0.55 (0.08) |
|  |  | + 1 SD | 0.02 (0.02) |  | [-0.03, 0.06] | 0.47 (0.07) |  | -0.01 (0.02) |  | [-0.06, 0.03] | 0.40 (0.12) |
| **N2 Amplitude** |  | - 1 SD | 0.65 (0.06) | *** | [0.53, 0.76] | -0.80 (0.24) |  | -0.19 (0.06) | ** | [-0.31, -0.08] | 0.35 (0.27) |
|  | Demand | Mean | 0.61 (0.04) | *** | [0.53, 0.69] | -0.67 (0.17) |  | -0.26 (0.04) | *** | [-0.34, -0.18] | 0.49 (0.19) |
|  |  | + 1 SD | 0.57 (0.06) | *** | [0.46, 0.69] | -0.54 (0.24) |  | -0.32 (0.06) | *** | [-0.44, -0.21] | 0.62 (0.27) |
|  |  | - 1 SD | 0.00 (0.02) |  | [-0.04, 0.05] | -0.80 (0.24) |  | 0.00 (0.04) |  | [-0.07, 0.07] | 0.35 (0.27) |
|  | Payoff | Mean | 0.01 (0.02) |  | [-0.02, 0.04] | -0.67 (0.17) |  | 0.03 (0.03) |  | [-0.02, 0.08] | 0.49 (0.19) |
|  |  | + 1 SD | 0.02 (0.02) |  | [-0.03, 0.06] | -0.54 (0.24) |  | 0.05 (0.04) |  | [-0.02, 0.12] | 0.62 (0.27) |
| **P3 Amplitude** |  | - 1 SD | -0.51 (0.09) | *** | [-0.70, -0.33] | 4.63 (0.23) |  | 0.18 (0.08) | ** | [0.02, 0.34] | 5.07 (0.25) |
|  | Demand | Mean | -0.60 (0.07) | *** | [-0.73, -0.47] | 4.58 (0.16) |  | 0.15 (0.06) | ** | [0.03, 0.26] | 5.10 (0.18) |
|  |  | + 1 SD | -0.69 (0.09) | *** | [-0.88, -0.51] | 4.54 (0.23) |  | 0.11 (0.08) |  | [-0.04, 0.27] | 5.13 (0.25) |
|  |  | - 1 SD | 0.08 (0.03) | ** | [0.02, 0.13] | 4.63 (0.23) |  | 0.08 (0.03) | ** | [0.02, 0.14] | 5.07 (0.25) |
|  | Payoff | Mean | 0.03 (0.02) |  | [-0.01, 0.07] | 4.58 (0.16) |  | 0.08 (0.02) | *** | [0.04, 0.12] | 5.10 (0.18) |
|  |  | + 1 SD | -0.02 (0.03) |  | [-0.07, 0.04] | 4.54 (0.23) |  | 0.08 (0.03) | ** | [0.02, 0.14] | 5.13 (0.25) |
| **Pupil Dilation** |  | - 1 SD | 0.74 (1.82) |  | [-2.84, 4.31] | 51.74 (3.68) |  | 8.31 (1.05) | *** | [6.26, 10.36] | 61.84 (4.27) |
|  | Demand | Mean | 0.17 (1.29) |  | [-2.36, 2.71] | 52.17 (2.60) |  | 8.57 (0.75) | *** | [7.09, 10.04] | 60.89 (3.01) |
|  |  | + 1 SD | -0.39 (1.83) |  | [-3.98, 3.2] | 52.61 (3.70) |  | 8.82 (1.05) | *** | [6.76, 10.88] | 59.94 (4.30) |
|  |  | - 1 SD | 0.14 (0.49) |  | [-0.81, 1.1] | 51.74 (3.68) |  | 1.89 (0.71) | ** | [0.49, 3.29] | 61.84 (4.27) |
|  | Payoff | Mean | 0.15 (0.35) |  | [-0.53, 0.82] | 52.17 (2.60) |  | 1.79 (0.51) | *** | [0.80, 2.79] | 60.89 (3.01) |
|  |  | + 1 SD | 0.15 (0.49) |  | [-0.80, 1.11] | 52.61 (3.70) |  | 1.70 (0.72) | ** | [0.29, 3.10] | 59.94 (4.30) |

*Note*. *N* ≥ 144. ****p* < .001, ***p* < .01, **p* < .05. CEI = cognitive effort investment.
