# Supplementary figures and images for: Cognitive effort investment: Does disposition become action?
Source: PLoS One. 2023 Aug 22;18(8):e0289428. doi: 10.1371/journal.pone.0289428 (PMC10443884; doi:10.1371/journal.pone.0289428)

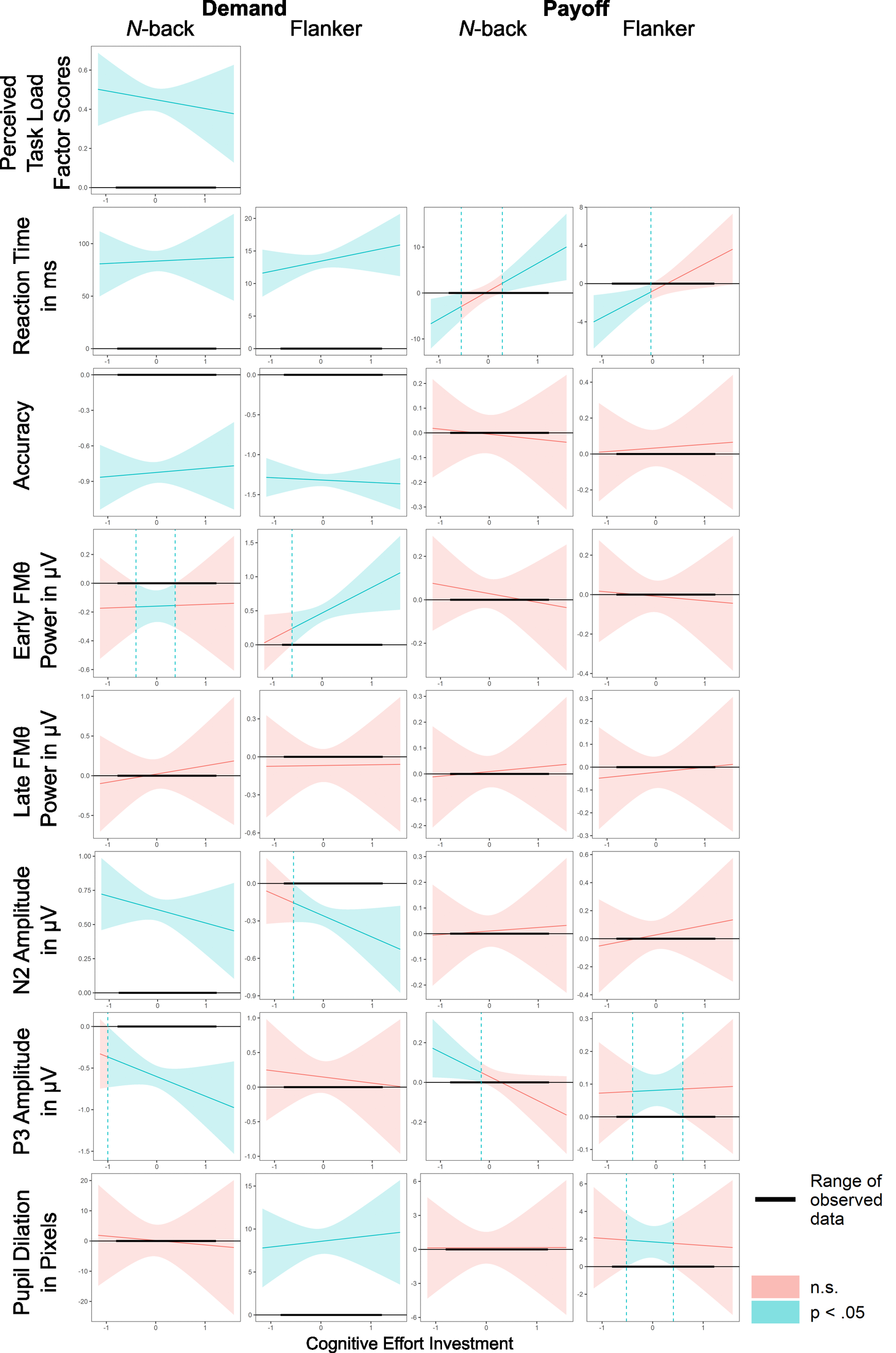

Supplement: S2 Fig — (TIF) [file pone.0289428.s004.tif]

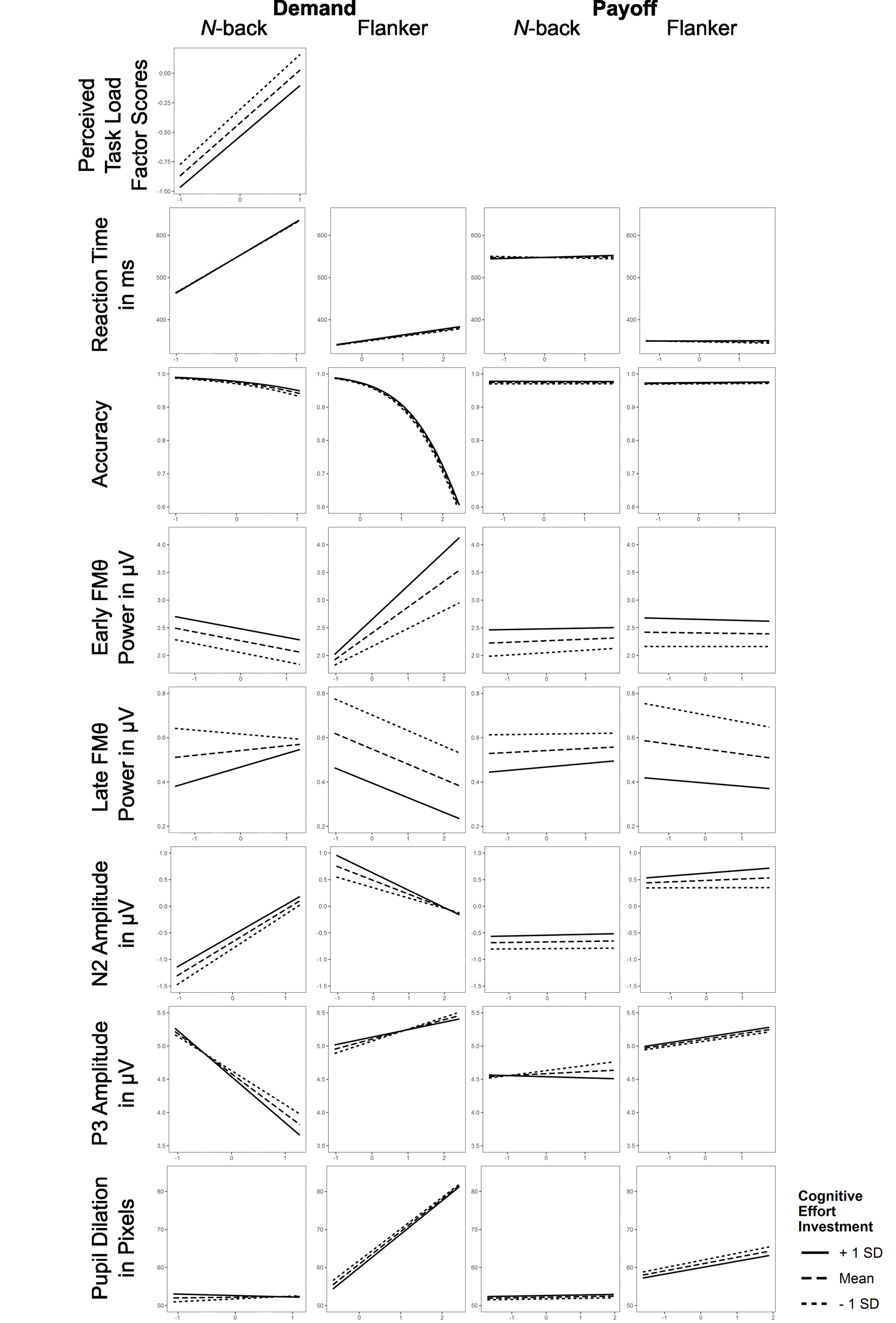

Supplement: S3 Fig — (TIF) [file pone.0289428.s005.tif]
