# Supplementary material for: Cognitive effort investment: Does disposition become action?
Source: PLoS One. 2023 Aug 22;18(8):e0289428. doi: 10.1371/journal.pone.0289428 (PMC10443884; doi:10.1371/journal.pone.0289428)
Supplement: Attachment — Submitted filename: Response to Reviewers.docx [file pone.0289428.s006.docx]

PONE-D-23-01823R1

Cognitive effort investment: Does disposition become action?

PLOS ONE

Response to Reviewers

We thank the reviewers and the editor again for their consideration of our manuscript and their thoughtful comments. We are pleased that we were able to convincingly answer all the issues raised by the first reviewer. In this revision, we have followed all of the advice given and hope that we have also been able to adequately address all of the issues raised by the second reviewer and the editor. Below, we explain our responses (author’s responses, AR) to each specific comment made by the reviewers (reviewer’s point, RP). We believe that the manuscript has considerably been improved by this process.

We highlighted all changes to the original version of the manuscript by red font in the file labeled 'Revised Manuscript with Track Changes' and provide an unmarked version labeled ‚Manuscript‘.

**Journal Requirements**

**EP1.** Please review your reference list to ensure that it is complete and correct. If you have cited papers that have been retracted, please include the rationale for doing so in the manuscript text, or remove these references and replace them with relevant current references. Any changes to the reference list should be mentioned in the rebuttal letter that accompanies your revised manuscript. If you need to cite a retracted article, indicate the article’s retracted status in the References list and also include a citation and full reference for the retraction notice.

**AR1.** The only reference we did not keep from our first submission is

Benjamini Y, Hochberg Y. Controlling the False Discovery Rate: A Practical and Powerful Approach to Multiple Testing. Journal of the Royal Statistical Society: Series B (Methodological). 1995;57(1):289-300. doi: 10.1111/j.2517-6161.1995.tb02031.x.

We deleted the sentence referring to this reference in response to RP1.11 (1^st^ revision). We apologize for not indicating this correctly in the resubmission.

Moreover, we carefully went through the reference list and edited where necessary.

**Reviewer #1**

The authors were very responsive and have addressed all issues raised during the revision process. I congratulate them on a fine manuscript!

Reviewer #2

I think the manuscript has been improved greatly. Some minor points could still profit from clarification, just to improve consistency and logical flow of an else really well written paper.

Page and line numbers below refer to the version with highlighted changes, and text snippets from the manuscript are included for easier review.

\###### Introduction: ######

**RP2.1.** p.3, l.33-34: ‘The general tendency of avoiding or at least reducing cognitive work – or effort – is called effort discounting [2].’

Comment: This definition of effort discounting is still imprecise (as it is used interchangeably with the ‘law of least mental effort’) and not exactly in line with the reference [2] provided here; see first round of review. It might be best to simply remove this sentence.

**AR2.1.** We thank the reviewer for bringing up this issue again and for clarifying the case where we have been imprecise. We refrained from removing the sentence because of its importance in the outline of our study, but specified both terms *effort discounting* and *law of less work* accordingly.

„In general, people prefer a less demanding behavioral option when it is rewarded as much as a more demanding one – but not all. Hull [1] ~~coined this principle~~ referred to the general tendency of avoiding or at least reducing energy expenditure or work as the *law of less work* ~~[1]~~. Yet, this principle of behavior is not only limited to physical work, but is true for cognitive work as well. ~~The general tendency of avoiding or at least reducing cognitive w~~ Work– or effort – even reduces the value associated with a given payoff – a phenomenon ~~is~~ called *effort discounting* [2]. While numerous studies …”

**RP2.2.** p.4, l.68-74: ‘(…) Westbrook et al. [6] experimentally showed that individuals with high need for cognition (i.e., a main indicator of cognitive effort investment) indicated a higher subjective value with demanding tasks than individuals with low need for cognition. Therefore, individuals with high levels in cognitive effort investment would be less likely to increase their effort based on increasing payoff, but rather based on increasing demand. In other words, when given equal demands, individuals with high compared to low levels of cognitive effort investment exert less effort.’

Comment: I appreciate the added section which this paragraph is a part of, with a good explanation of what is meant by efficient effort allocation and evidence presented for the intrinsic motivation of individuals with a high CEI disposition to perform effortful tasks. However, the conclusions in the last part do not strictly follow from the evidence presented. The evidence presented shows that those with high NFC seem to like demanding tasks more than those with low NFC. But this does neither (‘therefore’) indicate that the high-scorers would be less likely to increase their effort based on increasing payoff, nor (‘in other words’) that at equal demands high scorers would exert less effort. At least not directly – more evidence is needed to make this case and these predictions.

**AR2.2.** We restructured the entire paragraph and moved forward the supporting evidence as foundational evidence. By this, we now present our conclusions more strictly on the entire evidence rather than just the trait concept. This lead to the following revised paragraph:

“Do individuals with high cognitive effort investment actually invest effort more efficiently (i.e., dynamically according to task demands) in task processing and regardless of external incentives (e.g. payoff)? By definition these individuals are highly intrinsically motivated to perform effortful cognition [8], approach and enjoy cognitive challenges while reporting less difficulty and less negative emotions [11] and perform self-controlled behavior relatively effortlessly and unconsciously [12]. ~~For example~~ In line, Westbrook et al. [6] experimentally showed that individuals with high need for cognition (i.e., a main indicator of cognitive effort investment) indicated a higher subjective value with demanding tasks than individuals with low need for cognition. However, individuals with high need for cognition not only value demanding tasks more than those with low need for cognition, but also process these tasks with less effort as demonstrated by Mussel et al. [13]. They found that individuals with high levels of need for cognition, as a main component of cognitive effort investment, allocated their cognitive resources according to task demands, as indicated by frontal midline theta power (FMθ) in the electroencephalogram, while performing better in the harder task. This was not the case for individuals with low levels of need for cognition, who invested a considerable amount of resources even in easy tasks and performed comparably. In a study by Sandra and Otto [14], additional extrinsic motivation in terms of payoff did not add to the already high intrinsic motivation in individuals with high need for cognition to invest effort. They observed that individuals with low levels of need for cognition increased their amount of effort expenditure when facing higher payoff, whereas individuals with high need for cognition did not. ~~Therefore~~ According to the available evidence, individuals with high levels in cognitive effort investment would be less likely to increase their effort based on increasing payoff, but rather based on increasing demand. In other words, when given equal ~~demands~~ payoffs, individuals with high compared to low ~~levels of~~ cognitive effort investment invest effort according to task demands; whereas when given equal demands, they exert less effort, especially under low demand conditions. Thus, their performance would not benefit from payoff in terms of enhanced resource allocation (e.g. stronger increase in ~~frontal midline theta power (~~FMθ~~)~~ in the electroencephalogram) in order to improve performance (e.g. faster reaction times). Fig 2 hypothetically illustrates these interactions.

**Fig 2. Expected relation between actual effort investment and (A) demand and (B) payoff as a function of cognitive effort investment.**

~~First evidence that support this notion come from Mussel et al. [13] and Sandra and Otto [14]. The former found that individuals with high levels of need for cognition, as a main component of cognitive effort investment, allocated their cognitive resources according to task demands as indicated by FMθ in the electroencephalogram. This was not the case for individuals with low levels of need for cognition, who invested a considerable amount of resources even in easy tasks. The latter observed that individuals with low levels of need for cognition increased their amount of effort expenditure when facing higher payoff, whereas individuals with high need for cognition did not.~~”

**RP2.3.** p.6, l.134-136: ‘In line with our assumption that individuals with high levels of cognitive effort investment find task demands easier compared to individuals with low levels (…)’

Comment: At this point in the paper, this assumption appears a bit surprising as it has not been properly introduced and substantiated with evidence yet. While the authors earlier acknowledge the correlation between e.g. NFC and different cognitive ability/capacity measures, they have thus far focused on justifying why associations between NFC and demand- and payoff-related effort-adjustment are not due to differences in cognitive ability. But they have not clearly stated that they assume task demands will be perceived as easier by those scoring high on CEI.

**AR2.3.** We reduced the sentence to the main findings of Kramer et al.

„~~In line with our assumption that individuals with high levels of cognitive effort investment find task demands easier compared to individuals with low levels,~~ Kramer et al. [20] found a positive relation between need for cognition and performance (d’) for all *n*-back levels (i.e. 1-back, 2-back, 3-back), .14 < *r* < .22, in adolescents.“

\###### Discussion: ######

**RP2.4.** p. 44, l. 786-790: ‘In the present study, we were interested in the processes underlying this motivation to seek out effort. We focused on the main questions: (1) Is cognitive effort investment related to objective effort indices? (2) Does cognitive effort investment moderate the relation between effort indices and demand and payoff?’

Comment: Strictly speaking, there is no (direct) relation between the presented hypotheses and the interest in ‘the processes underlying this motivation to seek out effort’. Seeing the other revisions that were made, I am assuming the first sentence here was supposed to be changed as well.

**AR2.4.** Indeed, we missed to change this sentence and appreciate the reviewer’s careful reading. We changed the sentence accordingly.

„In the present study, we were interested in the ~~processes underlying this motivation to seek out effort~~ relationship between trait cognitive effort investment and actual effort investment during task processing. We focused on the main questions: (1) Is cognitive effort investment related to objective effort indices? (2) Does cognitive effort investment moderate the relation between effort indices and demand and payoff?“

**RP2.5.** p.45, l.801-804: ‘Hence, individuals with high levels of effortful self-control generally experience lower effort during task processing, which is consistent with the findings of de Ridder et al. [12] and strengthens our assumptions on individuals scoring high on cognitive effort investment.’

Comment: The last sentence sounds somewhat incomplete – which assumptions exactly does this strengthen?

**AR2.5.** We now provide more details on the findings of de Ridder et al. and on our assumption.

“Hence, individuals with high levels of effortful self-control generally experience lower effort during task processing~~, which~~. This is consistent with the meta-analytic findings of de Ridder et al. [12] that self-control correlates more strongly with automatic than controlled behavior, suggesting that individuals with high self-control tend to establish automatisms being unconscious and less effortful. Moreover, ~~and~~ it strengthens our assumption~~s~~ on individuals scoring high on cognitive effort investment to generally invest effort more efficiently.”
